# Modeling mixed boundary conditions in a Hilbert space with the complex variable boundary element method (CVBEM)

**DOI:** 10.1016/j.mex.2015.05.005

**Published:** 2015-05-20

**Authors:** Anthony N. Johnson, T.V. Hromadka

**Affiliations:** Department of Mathematical Sciences, United States Military Academy, 601 Swift Road, West Point, NY 10996, USA

**Keywords:** Complex variables, Hilbert space, Mixed boundary conditions, Stress, Approximate boundary, Complex variable boundary element method (CVBEM), Torsion, Least squares

## Abstract

The Laplace equation that results from specifying either the normal or tangential force equilibrium equation in terms of the warping functions or its conjugate can be modeled as a complex variable boundary element method or CVBEM mixed boundary problem. The CVBEM is a well-known numerical technique that can provide solutions to potential value problems in two or more dimensions by the use of an approximation function that is derived from the Cauchy Integral in complex analysis. This paper highlights three customizations to the technique.

• A least squares approach to modeling the complex-valued approximation function will be compared and analyzed to determine if modeling error on the boundary can be reduced without the need to find and evaluated additional linearly independent complex functions.
• The nodal point locations will be moved outside the problem domain.
• Contour and streamline plots representing the warping function and its complementary conjugate are generated simultaneously from the complex-valued approximating function.

## Graphical abstract

**Figure:**
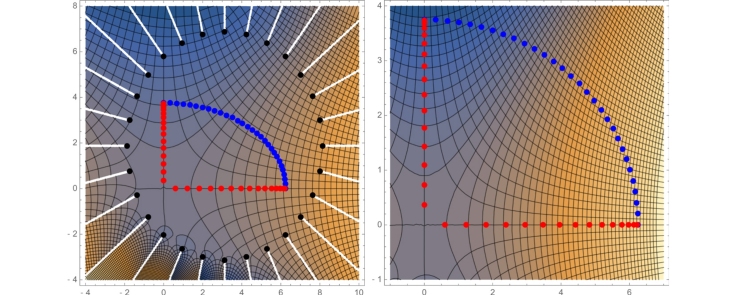

## Method details

The CVBEM has been developed by [1] for the solution of general problems involving Laplace or Poisson equations in multiple dimensions. Modeling begins with a simple closed contour of straight line segments. In a two dimensional complex plane, let Ω be bounded by a simple closed contour, Γ, such that

(1) $$\Gamma ={⋃}_{j=1}^{n}\Gamma_{j}.$$

**Fig. 1:**
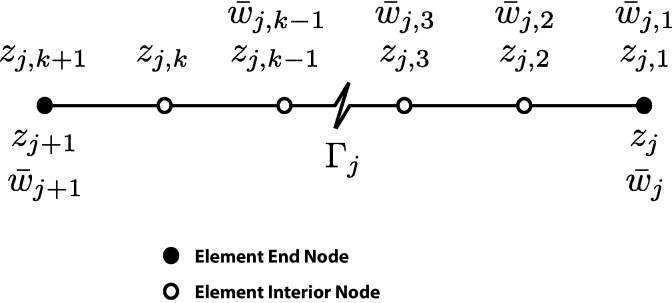
(*k* + 1)-Node boundary element Γ_*j*_ nodal definitions.

By defining (*k* + 1) equidistant nodal points in each Γ_*j*_ such that *z*_*j*,1_ and *z*_*j*,*k*+1_ are the endpoints of Γ_*j*_, the global nodal coordinates are related to local nodal coordinates by *z*_*j*,1_ = *z*_*j*_ and *z*_*j*,*k*+1_ = *z*_*j*+1,1_ = *z*_*j*+1_. Fig. 1 shows the global and local nodal numbering conventions. If one defines complex numbers *ω*_*ji*_ at each node *z*_*ji*_, then degree *k* complex polynomials $N_{j}^{k}(z)$ are uniquely defined on each boundary element Γ_*j*_.

A global trial function of order *k* is defined by

(2) $$G_{k}(z)=\sum_{j=1}^{n}\delta_{j}N_{j}^{k}(z),\;\;\;z\in \Gamma ,$$

where

$$\delta_{j}=\left\{\begin{array}{cc} 1 & z\in \Gamma ,\\ 0 & \mathrm{otherwise}; \end{array}\right)$$

*G*_*k*_(*z*) is continuous on Γ and

(3) $$\lim_{\max|\Gamma_{j}|\to 0}G_{k}(z)=\omega (z).$$

It is assumed that *ω*(*z*) is analytic on Γ ∪ Ω and that each ${\bar{\omega }}_{\mathrm{ji}}=\omega (z_{\mathrm{ji}})$.

**Fig. 2:**
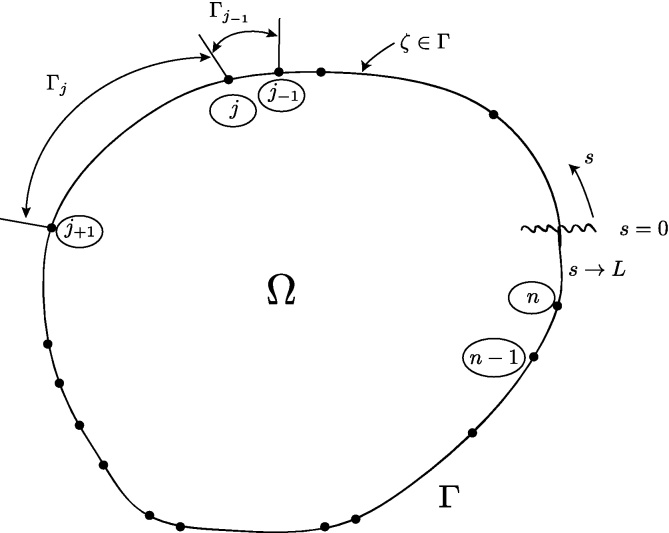
CVBEM [5].

**Fig. 3:**
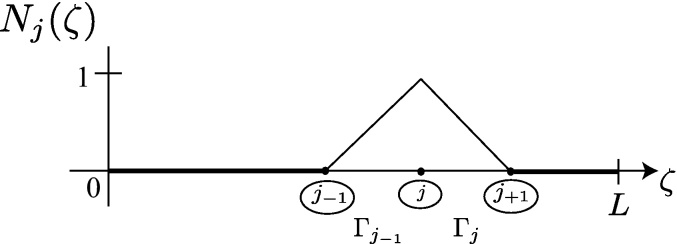
Linear interpolation basis functions, $N_{j}^{k}(\zeta )$.

Along the boundary Γ, or exterior to the problem domain union boundary, there are defined *n* nodal points. For development purposes, the *n* nodes are assumed defined on Γ [8]. Later, we will move the nodes outward away from the boundary to demonstrate an addition degree of freedom. The simple closed contour, Γ, in Fig. 2 is divided into *n* boundary elements, Γ_*j*−1_, Γ_*j*_,…,Γ_*n*_. For each boundary element, an interpolating polynomial will be used to create a piecewise continuous global interpolation function. In Fig. 2, the boundary, Γ, is “severed” at *s* = 0 and in the positive direction spans until *s* = *L*, the arc length of Γ. In Fig. 3, the boundary is “flattened” and the piecewise function presented. Here, *k* = 1 is chosen, and the complex polynomials $N_{j}^{k}(z)$ are uniquely defined as first order linear functions.

The piecewise function of Fig. 3 is

(4) $$N_{j}^{k}(\zeta )=\left\{\begin{array}{cc} \frac{\zeta -z_{j-1}}{z_{j}-z_{j-1}} & \zeta \in \Gamma_{j-1}\\ \frac{z_{j+1}-\zeta }{z_{j+1}-z_{j}} & \zeta \in \Gamma_{j}\\ 0 & \mathrm{otherwise} \end{array}\right)$$

Clearly, by its definition on Γ, $N_{j}^{k}(z)$ forms a basis as each boundary element produces an independent linear function. Note that the sum of the respective basis function terms is continuous on the boundary Γ for all *ζ* ∈ Γ. The basis function will be used to define a linear global trial function,

(5) $$G_{k}(\zeta )=\sum_{j=1}^{n}N_{j}^{k}(\zeta ){\bar{w}}_{j}$$

which is the sum of all nodal basis functions multiplied by a corresponding complex coefficient, ${\bar{w}}_{j}$, the nodal point *j* value of the function being approximated.

Consider the approximation function ${\hat{\omega }}_{k}(z)$ defined by

(6) $${\hat{\omega }}_{k}(z)=\frac{1}{2\pi i}\oint_{\Gamma }\frac{G_{k}(\zeta )d\zeta }{\zeta -z},\;z\notin \Gamma \;z\in \Omega .$$

From Eq. (2), the global trial function is substituted into the Cauchy Integral formula and integrated over a simply connected two-dimensional complex domain, Ω, with boundary, Γ, such that,

(7) $$\oint_{\Gamma }\frac{G_{k}(\zeta )d\zeta }{\zeta -z}=\oint_{\zeta }\frac{\sum \delta_{j}N_{j}^{k}(\zeta )d\zeta }{\zeta -z}=\sum \oint_{\Gamma_{j}}\frac{N_{j}^{k}(\zeta )d\zeta }{\zeta -z}.$$

On each Γ_*j*_, define a local coordinate system by

(8) $$\zeta_{j}=\zeta_{j}(s_{j})=z_{j}(z_{j+1}-z_{j})s_{j},\;\;\;\;\zeta_{j}\in \Gamma_{j},\;\;\;0\leq s_{j}\leq 1$$

It follows that

(9) $$\oint_{\Gamma_{j}}\frac{N_{j}^{k}(\zeta )d\zeta }{\zeta -z}=\int_{0}^{1}\frac{N_{j}^{k}(s_{j}){\mathrm{ds}}_{j}}{s_{j}-\gamma_{j}}$$

where $N_{j}^{k}(s_{j})=N_{j}^{k}(\zeta_{j}(s_{j}))$, and *γ*_*j*_ = (*z* − *z*_*j*_)/(*z*_*j*+1_ − *z*_*j*_) for *z* ∈ Γ.

Eq. (9) is solved by factoring (*s*_*j*_ − *γ*_*j*_) from $N_{j}^{k}(s_{j})$. Let $N_{j}^{k}(s_{j})$ be of the form

$$N_{j}^{k}(s_{j})=\sum_{i=0}^{k}{\mathbb{C}}_{j}^{i}s_{j}^{i},\;\;0\leq s_{j}^{i}\leq 1$$

where the ${\mathbb{C}}_{j}$ are complex constants in the form (*α* + *βi*). Division of $N_{j}^{k}(s_{j})$ by (*s*_*j*_ − *γ*_*j*_) gives

(10) $$\int_{0}^{1}\frac{N_{j}^{k}(s_{j}){\mathrm{ds}}_{j}}{s_{j}-\gamma_{j}}=R_{j}^{k}(z)+N_{j}^{k}(\gamma_{j})H_{j}$$

where $R_{j}^{k}(z)$ is a complex polynomial of degree *k* − 1, and

(11) $$H_{j}=\ln\frac{z_{j+1}-z}{z_{j}-z}=\ln\frac{d_{j+1}(z)}{d_{j}(z)}+i\theta_{j+1,j}(z).$$

**Fig. 4:**
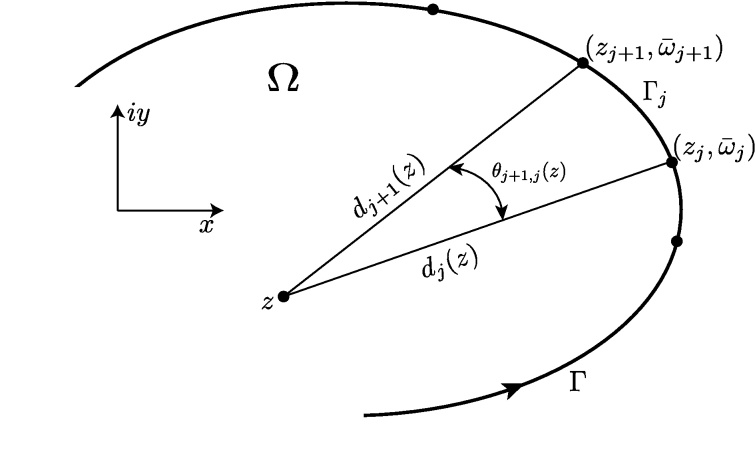
CVBEM linear trial function geometry.

Note that *d*_*j*_(*z*) = |*z*_*j*_ − *z*| and *θ*_*j*+1,*j*_(*z*) is the central angle between points *z*_*j*+1_, *z*_*j*_, and *z*. Fig. 4 shows the special case as *z* approaches Γ in the limit.

From Eqs. (6), (7), (9), (10), summation of the complex boundary element contributions from the *m* boundary element gives

(12) $$2\pi i{\hat{\omega }}_{k}(z)=\sum R_{j}^{k-1}(z)+\sum N_{j}^{k}(\gamma_{j})H_{j}$$

with $R^{k-1}(z)=\sum R_{j}^{k-1}(z)$, Eq. (10) simplifies to

(13) $${\hat{\omega }}_{k}(z)=\frac{1}{2\pi i}\left[R^{k-1}(z)+\sum N_{j}^{k}(\gamma_{j})H_{j}\right].$$

In Eq. (13), it is noted that the $N_{j}^{k}(\gamma_{j})$ have the form of the assumed shape functions on each *γ*_*j*_.

**Fig. 5:**
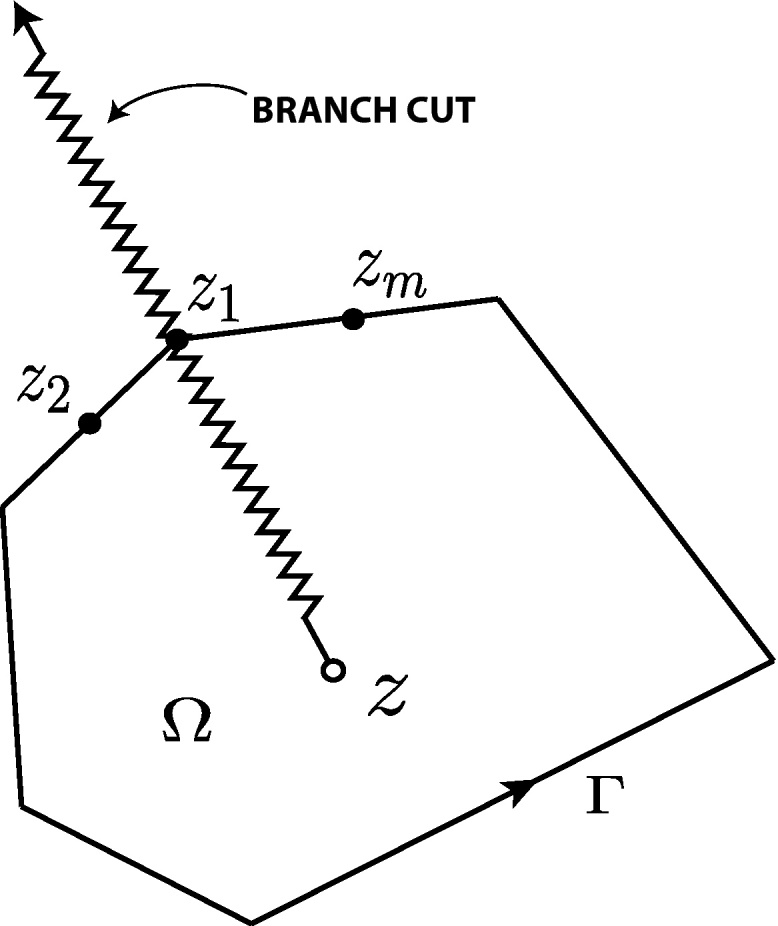
Branch cut of the function ln(*z* − *ζ*), *ζ* ∈ Γ.

Letting node *z*_1_ be on the branch cut of the complex logarithm function ln(*z* − *ζ*) such that *z* ∈ Ω and *ζ* ∈ Γ (see Fig. 5), then (13) can be expanded as

(14) $${\hat{\omega }}_{k}(z)=\frac{1}{2\pi i}R^{k-1}(z)-\frac{1}{2\pi i}\sum P_{j}^{k-1}(z-z_{j})\ln(z-z_{j})+N_{m}^{k}(z),$$

where $P_{j}^{k-1}$ is a polynomial of degree (*k* − 1) defined by

(15) $$P_{j}^{k-1}=\frac{\left(N_{j}^{k}(\gamma_{j})-N_{j-1}^{k}(\gamma_{j-1})\right)}{\left(z-z_{j}\right)}$$

and ln(*z* − *z*_*j*_) is the principal value of the logarithm function. From the continuity of *G*_*k*_(*ζ*), it is seen that at the nodal coordinate *z*_*j*_,

(16) $$N_{j}^{k}(\gamma_{j})-N_{j-1}^{k}(\gamma_{j-1})=0$$

and that (*z* − *z*_*j*_) is a factor as shown in (15). In (14), the $N_{m}^{k}$ term appears due to the circuit around the branch point of the multiple-valued function ln(*z* − *z*_*j*_).

Letting

$$R^{k}(z)=\frac{1}{2\pi i}R^{k-1}(z)+N_{m}^{k}(z),$$

then

(17) $${\hat{\omega }}_{k}(z)=R^{k}(z)-\frac{1}{2\pi i}\sum P_{j}^{k-1}(z-z_{j})\ln(z-z_{j}).$$

From (17), it is seen that ${\hat{\omega }}_{k}(z)$ is continuous over Ω and has removable singularities at each boundary element endpoint (nodal coordinate *z*_*j*_, *j* = 1, 2, 3, …, *m*).That is *R*^*k*^(*z*) and $P_{j}^{k-1}$ are continuous complex polynomials, and

$$\lim_{z\to z_{j}}(z-z_{j})\ln(z-z_{j})=0,\;i.e.\;\;{\hat{\omega }}_{k}(z_{j})=R^{k}(z_{j})$$

Note that since ${\hat{\omega }}_{k}(z)$ is analytic in Ω and ${\hat{\omega }}_{k}(z)=\hat{\phi }(z)+i\hat{\psi }(z)$ where $\hat{\phi }(z)$ and $\hat{\psi }(z)$ are two dimensional potential and stream functions which satisfy the Laplace equation exactly over Ω. By forcing the approximation values of ${\hat{\omega }}_{k}(z)$ to be arbitrarily close (within some *ϵ*) to the boundary-condition values of *ω*(*z*) on Γ, then it is guaranteed by the maximum modulus theorem that the approximation of *ω*(*z*) is bounded by $|\omega (z)-\hat{\omega }(z)|\leq \epsilon$, for all *z* ∈ Ω.

Because the CVBEM results in a two-dimensional function which is an exact solution to the governing partial differential equation on Ω, convergence of $\hat{\omega }(z)$ to *ω*(*z*) is then achieved on Ω ∪ Γ by forcing convergence on Γ. This is shown from (3), (6) by

(18) $$\lim_{\max|\Gamma_{j}|\to 0}\oint_{\Gamma }\frac{G_{k}(\zeta )d\zeta }{\zeta -z}=\oint_{\Gamma }\frac{\lim_{\max|\Gamma_{j}|\to 0}G_{k}(\zeta )d\zeta }{\zeta -z}=\oint_{\Gamma }\frac{\omega (\zeta )d\zeta }{\zeta -z}=2\pi i\omega (z).$$

The global trial function is continuous. Thus, $\hat{w}(z)$ is analytic in Ω, allowing $\hat{w}(z)$ to be used as an approximation function defined almost everywhere (“ae”) inside Ω as well as exterior to Ω. This characteristic separates the CVBEM from other approximations techniques. When solved, the CVBEM approximating integral becomes an approximating function of the form

(19) $$\hat{w}(z)=N_{0}(z)+N_{1}(z)+\sum_{j=1}^{n}{\mathbb{C}}_{j}(z-z_{j})\ln_{j}(z-z_{j})$$

with

$${\mathbb{C}}_{j}=\alpha_{j}+i\beta_{j}$$

where *α*_*j*_ and *β*_*j*_ are real constants to be determined, and

$$N_{0}(z)=(\alpha_{0}+i\beta_{0})\;\;\;\mathrm{and}\;\;\;N_{1}(z)=(\alpha_{-1}+i\beta_{-1})(x+\mathrm{iy})$$

where *α*_0_, *β*_0_, *α*_−1_, and *β*_−1_ are also real constants to be determined. The method steps [6] start by using the construct (*z* − *z*_*j*_) = *R*_*j*_*e*^*iθ*_*j*_^, defined at each node *j*, with location *z*_*j*_, Eq. (19) becomes

(20) $$\hat{w}(z)=N_{0}(z)+N_{1}(z)+\sum_{j=1}^{n}(\alpha_{j}+i\beta_{j})R_{j}e^{i\theta_{j}}\ln_{j}(R_{j}e^{i\theta_{j}}).$$

Using Euler's formula of *e*^*iθ*^ = (cos *θ* + *i* sin *θ*), the CVBEM approximation function becomes

(21) $$\hat{w}(z)=(\alpha_{0}+i\beta_{0})+(\alpha_{-1}+i\beta_{-1})(x+\mathrm{iy})+\sum_{j=1}^{n}(\alpha_{j}+i\beta_{j})R_{j}(\cos\theta_{j}+i\sin\theta_{j})\ln_{j}(R_{j}e^{i\theta_{j}}).$$

Further evaluation gives

(22) $$\ln_{j}(R_{j}e^{i\theta_{j}})=\ln_{j}(R_{j})+i\theta_{j}.$$

Combining terms from Eqs. (21), (22),

(23) $$\hat{w}(z)=(\alpha_{0}+i\beta_{0})+(\alpha_{-1}+i\beta_{-1})(x+\mathrm{iy})+\sum_{j=1}^{n}(\alpha_{j}R_{j}\cos\theta_{j}+i\alpha_{j}R_{j}\sin\theta_{j}+i\beta_{j}R_{j}\cos\theta_{j}+i^{2}\beta_{j}R_{j}\sin\theta_{j})(\ln_{j}(R_{j})+i\theta_{j}).$$

Collecting real and imaginary terms yield

(24) $$\hat{w}(z)=(\alpha_{0}+i\beta_{0})+(\alpha_{-1}+i\beta_{-1})(x+\mathrm{iy})+\sum_{j=1}^{n}(\alpha_{j}R_{j}(\ln_{j}(R_{j})\cos\theta_{j}-\theta_{j}\sin\theta_{j})-\beta_{j}R_{j}(\ln_{j}(R_{j})\sin\theta_{j}+\theta_{j}\cos\theta_{j})+i[\alpha_{j}R_{j}(\ln_{j}(R_{j})\sin\theta_{j}+\theta_{j}\cos\theta_{j})+\beta_{j}R_{j}(\ln_{j}(R_{j})\cos\theta_{j}-\theta_{j}\sin\theta_{j})]).$$

We can now separate the approximation function into real, $\hat{\phi }$, and imaginary, $\hat{\psi }$, parts:

(25) $$\hat{w}(z)=\hat{\phi }(z)+i\hat{\psi }(z)$$

where the potential functions, or real parts, are given by

(26) $$\hat{\phi }(z)=\alpha_{0}+(\alpha_{-1}x-\beta_{-1}y)+\hat{\Phi }(z)$$

for

(27) $$\hat{\Phi }(z)=\sum_{j=1}^{n}(\alpha_{j}R_{j}(\ln_{j}(R_{j})\cos\theta_{j}-\theta_{j}\sin\theta_{j})-\beta_{j}R_{j}(\ln_{j}(R_{j})R_{j}\sin\theta_{j}+\theta_{j}\cos\theta_{j}))$$

and where the stream functions, or imaginary parts, are given by

(28) $$\hat{\psi }(z)=\beta_{0}+(\beta_{-1}x+\alpha_{-1}y)+\hat{\Psi }(z)$$

for

(29) $$\hat{\Psi }(z)=\sum_{j=1}^{n}(\alpha_{j}R_{j}(\ln_{j}(R_{j})\sin\theta_{j}+\theta_{j}\cos\theta_{j})+\beta_{j}R_{j}(\ln_{j}(R_{j})\cos\theta_{j}-\theta_{j}\sin\theta_{j})).$$

Recall that ln _*j*_ includes the effect of the nodal point logarithmic branch cut rotations.

## Matrix formulation and model strategy

After the real and imaginary parts of the CVBEM approximation equation have been developed, the next step is to find the constants, *α*_*n*_, and *β*_*n*_, in the $\hat{\phi }$ and $\hat{\psi }$ functions. The two numerical modeling strategies investigated and subsequently compared for both accuracy and efficiency in this paper are collocation and a least squares approach in a Hilbert space. Each has advantages and disadvantages. Our goal is to highlight which modeling strategy works best for mixed boundary value problems with irregular boundaries.

### Collocation

For collocation, the approach is to use the CVBEM by juxtaposing Eq. (19) at each nodal point specified on Γ (in the limit as *z* approaches Γ from inside Ω). Generally, only one nodal value of either *ϕ* or *ψ* is known at each nodal point [4]. Consequently for *m* nodes specified on Γ, there are 2*m* values of {*ϕ*_*j*_, *ψ*_*j*_}, and only *m* nodal values are known as boundary conditions. Collocating Eq. (19) at each node generates *m* equations for the *m* unknown nodal values. The resulting *m* × *m* matrix system results in the determination of the $\hat{\omega }(z)$ approximator, which is analytic in Ω. That is, $\hat{\omega }(z)$ operates on the 2*m* nodal values {*ϕ*_*j*_, *ψ*_*j*_} and the coordinate *z*. Next is to develop an analytic continuation of the $\hat{\omega }(z)$ approximator which matches the specified and computed 2*m* nodal values of Γ. The advantage of using Eq. (19) is that the Cauchy integral of Eq. (6) has the property that $\hat{\omega }(z)$ only has non-zero value in Ω ∪ Γ. That is,

(30) $$\hat{\omega }(z)=\left\{\begin{array}{cc} \hat{\omega }(z), & z\in \Omega \cup \Gamma ,\\ 0 & z\notin \Omega \cup \Gamma . \end{array}\right)$$

Consider the real portion of the CVBEM with one node for collocation point *k*. The resulting equation from Eq. (26) is:

(31) $${\hat{\phi }}_{k}(z)={\hat{\phi }}_{k}(x_{k}+{\mathrm{iy}}_{k})=\alpha_{0}+\alpha_{1}x_{k}-\beta_{1}y_{k}+\alpha_{2}p_{1,k}-\beta_{2}q_{1,k}$$

where

(32) $$\begin{array}{cc} p_{n,k}=R_{n,k}(\ln(R_{n,k})\cos\theta_{n,k}-\theta_{n,k}\sin\theta_{n,k} & \\ q_{n,k}=R_{n,k}(\ln(R_{n,k})\sin\theta_{n,k}-\theta_{n,k}\cos\theta_{n,k} & \end{array}.$$

Evaluate the above with the five necessary potential collocation points on the problem boundary. This will result in five linearly independent equations for five collocation points on Γ,

(33) $$\begin{array}{cc} {\hat{\phi }}_{k}(z)={\hat{\phi }}_{k}(x_{1}+{\mathrm{iy}}_{1})=\alpha_{0}+\alpha_{1}x_{1}-\beta_{1}y_{1}+\alpha_{2}p_{1,1}-\beta_{2}q_{1,1} & \\ {\hat{\phi }}_{k}(z)={\hat{\phi }}_{k}(x_{2}+{\mathrm{iy}}_{2})=\alpha_{0}+\alpha_{1}x_{2}-\beta_{1}y_{2}+\alpha_{2}p_{1,2}-\beta_{2}q_{1,2} & \\ {\hat{\phi }}_{k}(z)={\hat{\phi }}_{k}(x_{3}+{\mathrm{iy}}_{3})=\alpha_{0}+\alpha_{1}x_{3}-\beta_{1}y_{3}+\alpha_{2}p_{1,3}-\beta_{2}q_{1,3} & \\ {\hat{\phi }}_{k}(z)={\hat{\phi }}_{k}(x_{4}+{\mathrm{iy}}_{4})=\alpha_{0}+\alpha_{1}x_{4}-\beta_{1}y_{4}+\alpha_{2}p_{1,4}-\beta_{2}q_{1,4} & \\ {\hat{\phi }}_{k}(z)={\hat{\phi }}_{k}(x_{5}+{\mathrm{iy}}_{5})=\alpha_{0}+\alpha_{1}x_{5}-\beta_{1}y_{5}+\alpha_{2}p_{1,5}-\beta_{2}q_{1,5} & \end{array}.$$

The matrix system to be solved is simply *Ax* = *b*, where *A* is a coefficient matrix and *b* are the known potential values at each of the collocation points:

(34) $$\left[\begin{array}{c} {\hat{\phi }}_{1}\\ {\hat{\phi }}_{2}\\ {\hat{\phi }}_{3}\\ {\hat{\phi }}_{4}\\ {\hat{\phi }}_{5} \end{array}\right]=\left[\begin{array}{cccccc} 1 & x_{1} & -y_{1} & p_{1,1} & -q_{1,1} & \\ 1 & x_{2} & -y_{2} & p_{1,2} & -q_{1,2} & \\ 1 & x_{3} & -y_{3} & p_{1,3} & -q_{1,3} & \\ 1 & x_{4} & -y_{4} & p_{1,4} & -q_{1,4} & \\ 1 & x_{5} & -y_{5} & p_{1,5} & -q_{1,5} & \end{array}\right]\left[\begin{array}{c} \alpha_{0}\\ \alpha_{1}\\ \beta_{1}\\ \alpha_{2}\\ \beta_{2} \end{array}\right].$$

**Fig. 6:**
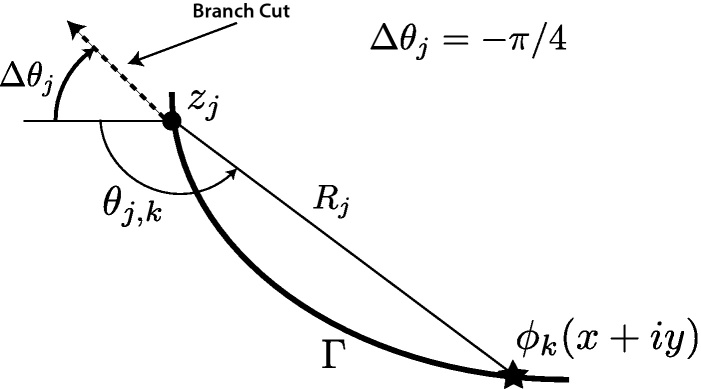
Solving for *R* and *θ* in Eq. (32).

Once the system has been configured, substitute the coordinates of the collocation points into the second and third column of the coefficient matrix. It is also necessary to calculate the radius and angle of the collocation points from the singleton node, (see Fig. 6).

The final step is to solve the matrix system. Once these values are known, they can be substituted back into the original equation for $\hat{\phi }(z)$. The $\hat{\phi }$ function can now be used to approximate all the potential values within the problem domain. Collocation can also be used in the same way to solve for the streamline equation, $\hat{\psi }(z)$. The most significant change is that instead of solving for *α*_0_, one must solve for *β*_0_.

### Least squares

For least squares, the approach is similar except now we will use the evaluation points on the known boundary to create an overdetermined system. From Fig. 1, the values, ${\bar{\omega }}_{j}(z)$ are known for either ${\bar{\phi }}_{j}(z)$ or ${\bar{\psi }}_{j}(z)$ or both. We can now create a vector of measurements along the boundary such that values ${\bar{\omega }}_{1}\ldots {\bar{\omega }}_{m}$ are available. Using *n* basis functions (*z* − *z*_*j*_) ln(*z* − *z*_*j*_), we wish to find the complex coefficients, ${\mathbb{C}}_{j}(z)$ such that the approximating function

(35) $$\hat{\omega }(z)=\sum_{j=1}^{n}{\mathbb{C}}_{j}(z)(z-z_{j})\ln(z-z_{j})$$

minimizes the sum of squares such that

(36) $$F({\mathbb{C}}_{1},{\mathbb{C}}_{2},\cdots ,{\mathbb{C}}_{n})=\sum_{j=1}^{m}{\left(b_{k}-\sum_{j=1}^{n}{\mathbb{C}}_{j}(z)(z-z_{j})\ln(z-z_{j})\right)}^{2}=\min.$$

The need for using Eq. (19) becomes apparent when determining the approximate boundary which is associated with the CVBEM approximator functions, ${\hat{\omega }}_{k}(z)$.

### Approximate boundary

In applying the CVBEM to mixed boundary problems, it is necessary to develop an approximate boundary, $\hat{\Gamma }$, upon which ${\hat{\omega }}_{k}(z)$ satisfies the problem boundary conditions. Engineering problems related to stress and strain are adequate candidates for CVBEM analysis. For stress-free boundary conditions, $\hat{\Gamma }$ is the collection of points defined by

(37) $$\hat{\Gamma }=\left\{z:\hat{\phi }(z)=\frac{1}{2}|z^{2}|\right\},$$

where $\hat{w}(z)=\hat{\phi }(z)+i\hat{\psi }(z)$. Also |*z*|^2^ = *x*^2^ + *y*^2^ where |*z*| is measured from a selected central point in Ω. If $\hat{\Gamma }$ coincides with Γ, then necessarily $\hat{\omega }(z)=\omega (z)$ on Ω ∪ Γ. The utility of the approximate boundary concept is in the evaluation of the approximation error. Instead of the analysis of abstract error quantities, the goodness of approximation is determined by visually inspecting the closeness-of-fit between $\hat{\Gamma }$ and Γ. In those regions, where $\hat{\Gamma }$ deviates substantially from Γ, additional evaluation points are placed to reduce the approximation errors from using the selected shape functions.

## Analysis and numerical results

**Fig. 7:**
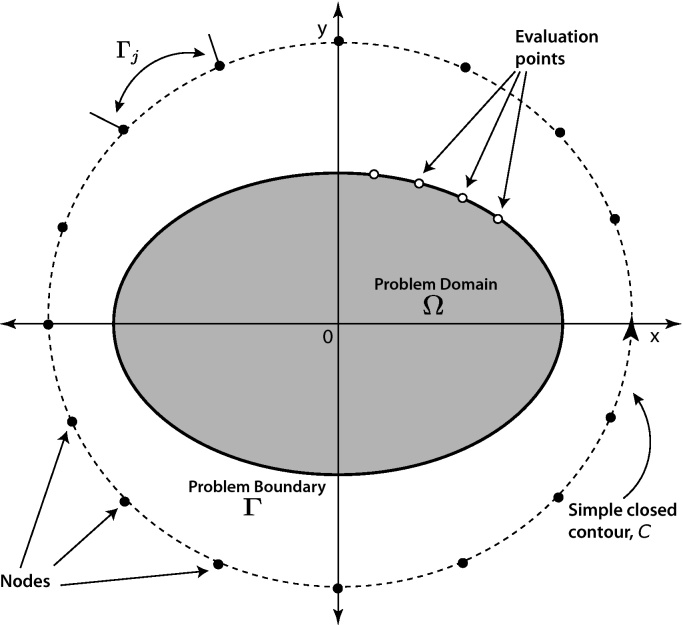
Nodes placed outside the domain highlight an addition degree of freedom. Axis symmetry allows simplification of the problem to the first quadrant.

As an example of the complex variable boundary element method consider the twisting behavior of a homogeneous, isotropic shaft of an arbitrary, but uniform, cross section that is fixed at one end and subjected to a twisting couple at the other end. If the force and deformation behavior is of interest at some location somewhat removed from either end, then the stress and strain characteristics of the cross section as depicted in Fig. 7 are described by either of the following equations [3]:

(38) $$\frac{\partial^{2}\psi (x,y)}{\partial x^{2}}+\frac{\partial^{2}\psi (x,y)}{\partial y^{2}}=0,$$

(39) $$\frac{\partial^{2}\phi (x,y)}{\partial x^{2}}+\frac{\partial^{2}\phi (x,y)}{\partial y^{2}}=0.$$

The quantity *ψ*(*x*, *y*) is the warping function of the cross-section whereas *ϕ*(*x*, *y*) is the conjugate of *ψ*(*x*, *y*). If the warping function is known over the cross-section, then the out-of-plane warping displacement and the in-plane shear stresses can be calculated from the expressions

(40) $$\omega =\theta \psi (x,y),\;\tau_{\mathrm{xz}}=\mu \theta \left(\frac{\partial \psi (x,y)}{\partial x}-y\right),\;\tau_{\mathrm{yz}}=\mu \theta \left(\frac{\partial \psi (x,y)}{\partial y}-x\right).$$

In the above expressions *θ* is the angle of the twist per unit length, *μ* is the shear modulus, and *x*, *y* denote the coordinates of a point located from the center of twist. Furthermore, it should be noted that *z* represents a coordinate axis and should not be confused with the complex variable *z* = *x* + *iy*. If, on the other hand, the problem is posed in terms of the complementary function *ϕ*(*x*, *y*) then the shear stresses are determined from

(41) $$\tau_{\mathrm{xz}}=\mu \theta \left(\frac{\partial \phi (x,y)}{\partial y}-y\right),\;\;\;\tau_{\mathrm{yz}}=\mu \theta \left(-\frac{\partial \phi (x,y)}{\partial x}+x\right).$$

While the form of Eqs. (38), (31) are identical, a solution strategy emerges depending on the manner in which the boundary conditions are specified. If the boundary condition of zero normal stress around the perimeter is posed, then a Neumann boundary condition, i.e. specified normal derivative, best describes the problem. In such a case the nonuniform torsion problem is best posed in terms of the warping function, *ψ*(*x*, *y*). If on the other hand, the problem is best posed in terms of zero shear around the perimeter, then a Dirichlet boundary condition, i.e. specified functions, best describe the problem. In such a case the problem is best posed in terms of the complementary function, *ϕ*(*x*, *y*). While either solution method is well adapted for solid shafts, it is generally more convenient to operate directly with the warping function, *ψ*(*x*, *y*), rather than its conjugate, *ϕ*(*x*, *y*), for hollow cross-sections.

The following example is used to analyze the CVBEM with established solutions [7] for shaft cross-sections of smooth and sharp corner profiles. Consider the torsion of a solid elliptical cross section with major axis *a* and minor axis *b*. The shear–stress-free boundary condition can be expressed in terms of the conjugate function *ϕ*(*x*, *y*) expressed on the boundary as

(42) $$\phi (x,y)=\frac{1}{2}(x^{2}+y^{2})$$

The conjugate function *ϕ*(*x*, *y*) as well as the shear stresses can be shown to be

(43) $$\phi (x,y)=\frac{1}{2}(x^{2}+y^{2})-a^{2}b^{2}\left(\frac{x^{2}}{a^{2}}+\frac{y^{2}}{b^{2}}-1\right){\left(a^{2}+b^{2}\right)}^{-1}$$

(44) $$\tau_{\mathrm{xz}}=-\mu \theta \frac{2{\mathrm{ya}}^{2}}{\left(a^{2}+b^{2}\right)}$$

(45) $$\tau_{\mathrm{yz}}=-\mu \theta \frac{2{\mathrm{xb}}^{2}}{\left(a^{2}+b^{2}\right)}$$

**Fig. 8:**
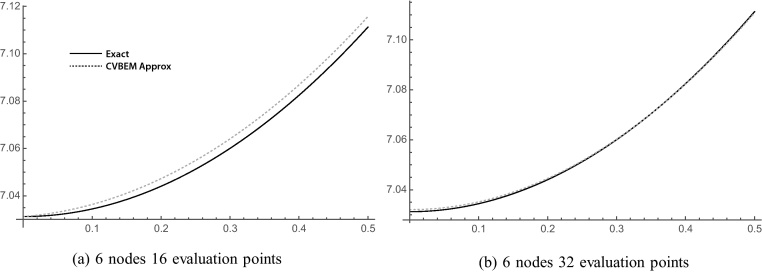
Approximate boundary for 6 node collocation and least squares models taken along a very small portion of the elliptical boundary.

**Fig. 9:**
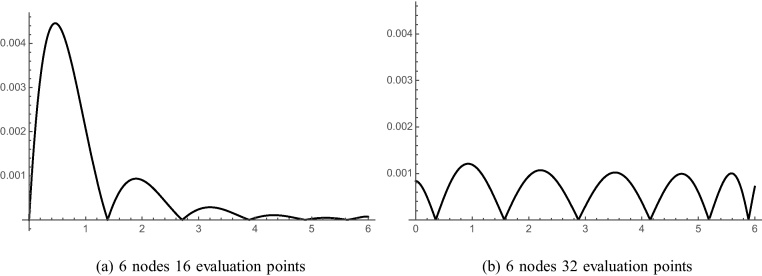
CVBEM relative error along the quarter elliptical section boundary computed as $\left[\hat{\phi }(x,y)-\phi (x,y)]/\phi (x,y\right)$ where $\hat{\phi }(x,y)$ is the CVBEM approximate solution and *ϕ*(*x*, *y*) is the exact solution.

**Fig. 10:**
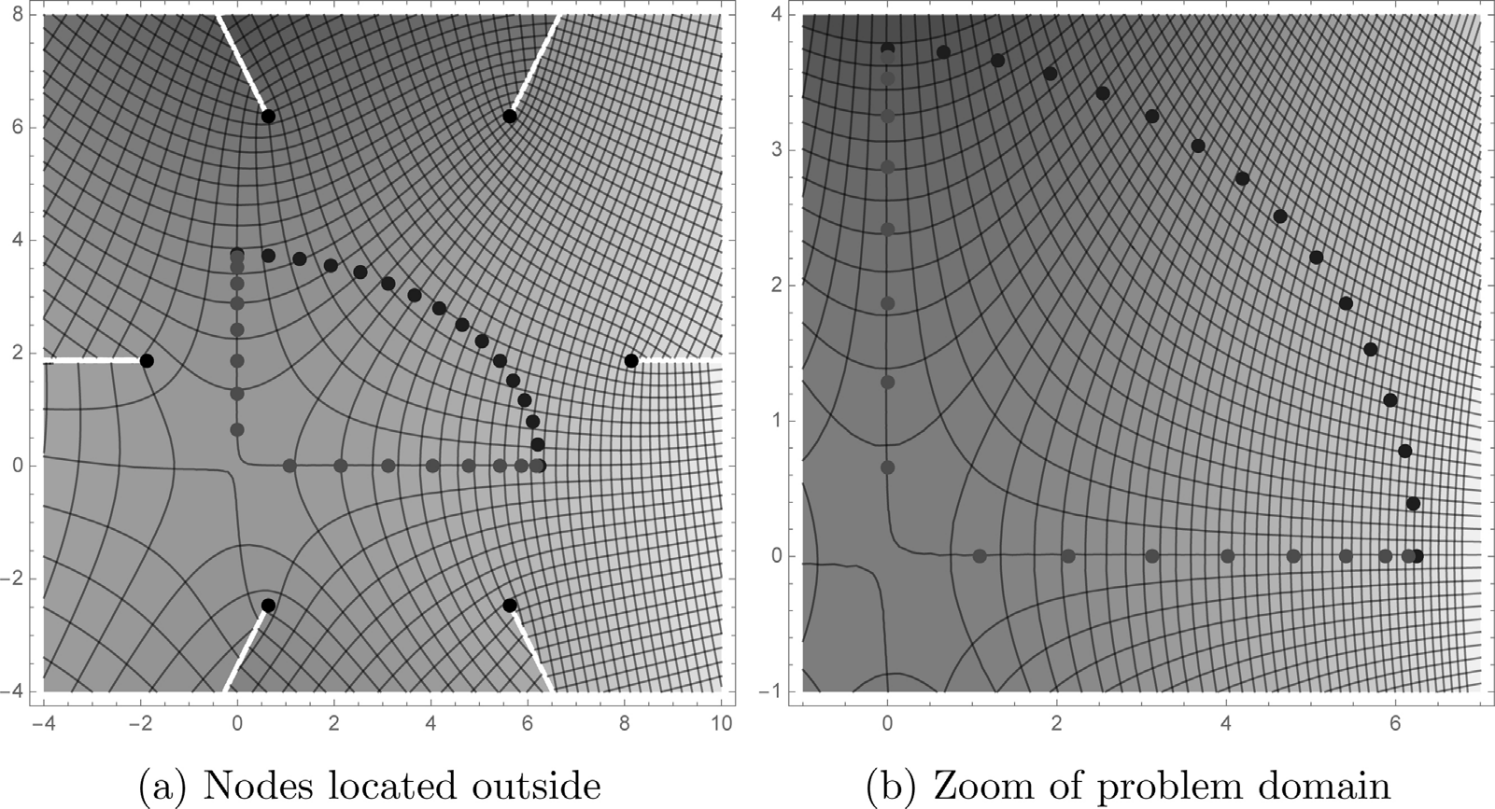
Simultaneous contour plots of the streamline and potential functions. The two real-valued functions, $\hat{\phi }(z)$ and $\hat{\psi }(z)$, relate stress and strain within the domain and are the real and complex components of the complex-valued approximating function $\hat{\omega }(z)=\hat{\phi }(z)+i\hat{\psi }(z)$.

**Fig. 11:**
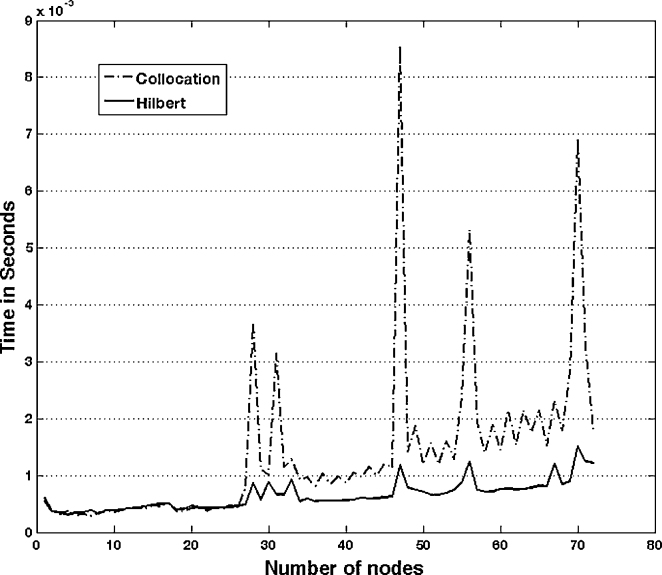
Time comparison for collocation versus least squares method.

Fig. 8 displays the approximate boundary for 6 node (a) collocation and (b) least squares models taken along a very small portion of the elliptical boundary. The deviation of Fig. 8a is due to error. Fig. 8b shows some error, but nearly as much. Fig. 9 shows the relative error for the 6-node model, respectively. The CVBEM relative error along the quarter elliptical section boundary is computed as $\left[\hat{\phi }(x,y)-\phi (x,y)]/\phi (x,y\right)$ where $\hat{\phi }(x,y)$ is the CVBEM approximate solution and *ϕ*(*x*, *y*) is the exact solution. The quarter model of Fig. 7 was chosen to take advantage of the problem symmetry and to demonstrate the imposition of *ϕ* boundary conditions along the exterior curved edge and *ψ* along the interior straight edge which is the side extending from the origin to the point (*a*, 0) and the line extending from the origin to the point (0, *b*) where *a* and *b* are 6.25 and 3.75 respectively. Table 1 summarizes the exact and computed warping function and shear–stress values at points in Ω using the collocation method. Table 2 demonstrates the exact and warping function calculation using least squares for the same number of basis functions, which are the nodes. The graphical depiction of the CVBEM in Fig. 10 uses computer programs Matlab, Mathematica, and MATLink to model the mixed boundary problem. Fig. 11 is a time analysis of the efficiency of collocation compared to least squares.

## Additional information

The complex variable boundary element method (CVBEM) has been shown to be a mathematically sound approach for modeling two-dimensional potential problems [2]. The foundations of the CVBEM method rests in complex variable theory, namely, the Cauchy integral formula. It tells us that if a function, *ω*, is analytic within and on a simple closed contour, Γ, then the values of *ω* interior to Γ are completely determined by the values on Γ. In other words, to determine the values within the domain, Ω, one simple need only know the values on the boundary. Thus, an approximation function is developed that is analytic over the problem domain (i.e. possesses derivatives of all orders) and has both real and imaginary parts which exactly solve the Laplace equation within Ω.

Error analysis is an area that makes the CVBEM particularly appealing. It is unique among other numerical methods in that the CVBEM approximating function can be evaluated directly to analyze the error of the approximation. This is because the CVBEM develops an exact representation of the modeling error by the determination of an ‘approximate boundary’ where the CVBEM approximation exactly satisfies the boundary conditions. That is, the approximate boundary is the locus of points where the CVBEM approximation meets the boundary condition values. This approach is in stark contrast to other numerical methods in their analysis of error. Two sources of error are primary in most numerical methods. The two sources consists of error that results from the approximation in solving the governing equation, and error that result in solving the boundary conditions continuously. Popular numerical methods such as finite elements (FEM) and finite differences (FDM) generate both types of errors in modeling potential problems. Model accuracy is usually estimated by comparing the change in results by increasing the number of nodal points. By this the analyst is seeking convergence by showing that the result is both stable and consistent.

## Figures and Tables

**Table 1:** CVBEM 6-node model collocation method.

x	y	Exact	CVBEM(*ϕ*)	Error(%)	Shear(*τ*_*xz*_)	CVBEM(*τ*_*xz*_)	Error(%)
1	1	10.3401	10.2159	1.201	−1.47059	−1.43278	2.571 * 10^−2^
1	2	9.63419	9.55065	8.671 * 10^−1^	−2.94118	−2.89735	1.49 * 10^−2^
1	3	8.45772	8.4218	4.248 * 10^−1^	−4.41176	−4.36002	1.173 * 10^−2^
1	4	6.81066	6.82913	2.712 * 10^−1^	−5.88235	−5.82853	9.149 * 10^−3^
1	5	4.69301	4.74572	1.123	−7.35294	−7.35907	8.335 * 10^−4^
2	1	11.046	10.9448	9.16 * 10^−1^	−1.47059	−1.43203	2.622 * 10^−2^
2	2	10.3401	10.2775	6.053 * 10^−1^	−2.94118	−2.90204	1.331 * 10^−2^
2	3	9.1636	9.14085	2.483 * 10^−1^	−4.41176	−4.37161	9.101 * 10^−3^
2	4	7.51654	7.53193	2.047 * 10^−1^	−5.88235	−5.84846	5.761 * 10^−3^
2	5	5.3989	5.43676	7.013 * 10^−1^	−7.35294	−7.3463	9.031 * 10^−4^
3	1	12.2224	12.1451	6.331 * 10^−1^	−1.47059	−1.43723	2.268 * 10^−2^
3	2	11.5165	11.4729	3.794 * 10^−1^	−2.94118	−2.90729	1.152 * 10^−2^
3	3	10.3401	10.3304	9.354 * 10^−2^	−4.41176	−4.37762	7.739 * 10^−3^
3	4	8.69301	8.71804	2.878 * 10^−1^	−5.88235	−5.84635	6.12 * 10^−3^
3	5	6.57537	6.64249	1.021	−7.35294	−7.29947	7.272 * 10^−3^
4	1	13.8695	13.8153	3.91 * 10^−1^	−1.47059	−1.44091	2.018 * 10^−2^
4	2	13.1636	13.1385	1.904 * 10^−1^	−2.94118	−2.91248	9.758 * 10^−3^
4	3	11.9871	11.9904	2.741 * 10^−2^	−4.41176	−4.38371	6.358 * 10^−3^
4	4	10.3401	10.3715	3.035 * 10^−1^	−5.88235	−5.85362	4.884 * 10^−3^
4	5	8.22243	8.28744	7.906 * 10^−1^	−7.35294	−7.30922	5.946 * 10^−3^
5	1	15.9871	15.9574	1.862 * 10^−1^	−1.47059	−1.4448	1.754 * 10^−2^
5	2	15.2813	15.2755	3.74 * 10^−2^	−2.94118	−2.9181	7.845 * 10^−3^
5	3	14.1048	14.1217	1.197 * 10^−1^	−4.41176	−4.38984	4.969 * 10^−3^
5	4	12.4577	12.4938	2.897 * 10^−1^	−5.88235	−5.86819	2.408 * 10^−3^
5	5	10.3401	10.3757	3.449 * 10^−1^	−7.35294	−7.37614	3.155 * 10^−3^

**Table 2:** CVBEM 6-node model least squares method.

x	y	Exact	CVBEM(*ϕ*)	Error(%)	Shear(*τ*_*xz*_)	CVBEM(*τ*_*xz*_)	Error(%)
1	1	10.3401	10.3158	2.352 * 10^(^ − 1)	−1.47059	−1.46325	4.989 * 10^(^ − 3)
1	2	9.63419	9.61789	1.692 * 10^(^ − 1)	−2.94118	−2.93276	2.862 * 10^(^ − 3)
1	3	8.45772	8.45026	8.825 * 10^(^ − 2)	−4.41176	−4.40243	2.115 * 10^(^ − 3)
1	4	6.81066	6.81127	8.974 * 10^(^ − 3)	−5.88235	−5.87834	6.822 * 10^(^ − 4)
1	5	4.69301	4.67955	2.868 * 10^(^ − 1)	−7.35294	−7.39894	6.256 * 10^(^ − 3)
2	1	11.046	11.0267	1.745 * 10^(^ − 1)	−1.47059	−1.46358	4.764 * 10^(^ − 3)
2	2	10.3401	10.328	1.166 * 10^(^ − 1)	−2.94118	−2.93367	2.552 * 10^(^ − 3)
2	3	9.1636	9.15949	4.484 * 10^(^ − 2)	−4.41176	−4.40323	1.935 * 10^(^ − 3)
2	4	7.51654	7.5224	7.794 * 10^(^ − 2)	−5.88235	−5.86995	2.109 * 10^(^ − 3)
2	5	5.3989	5.42563	4.951 * 10^(^ − 1)	−7.35294	−7.31646	4.962 * 10^(^ − 3)
3	1	12.2224	12.2078	1.199 * 10^(^ − 1)	−1.47059	−1.46453	4.117 * 10^(^ − 3)
3	2	11.5165	11.5081	7.308 * 10^(^ − 2)	−2.94118	−2.93486	2.146 * 10^(^ − 3)
3	3	10.3401	10.3379	2.071 * 10^(^ − 2)	−4.41176	−4.40548	1.424 * 10^(^ − 3)
3	4	8.69301	8.69823	6.004 * 10^(^ − 2)	−5.88235	−5.8723	1.71 * 10^(^ − 3)
3	5	6.57537	6.60224	4.087 * 10^(^ − 1)	−7.35294	−7.31058	5.761 * 10^(^ − 3)
4	1	13.8695	13.859	7.583 * 10^(^ − 2)	−1.47059	−1.46465	4.036 * 10^(^ − 3)
4	2	13.1636	13.1589	3.61 * 10^(^ − 2)	−2.94118	−2.93558	1.904 * 10^(^ − 3)
4	3	11.9871	11.9871	1.129 * 10^(^ − 4)	−4.41176	−4.40877	6.778 * 10^(^ − 4)
4	4	10.3401	10.3374	2.601 * 10^(^ − 2)	−5.88235	−5.89386	1.957 * 10^(^ − 3)
4	5	8.22243	8.19223	3.673 * 10^(^ − 1)	−7.35294	−7.39874	6.229 * 10^(^ − 3)
5	1	15.9871	15.9812	3.693 * 10^(^ − 2)	−1.47059	−1.46561	3.386 * 10^(^ − 3)
5	2	15.2813	15.2803	6.438 * 10^(^ − 3)	−2.94118	−2.93539	1.966 * 10^(^ − 3)
5	3	14.1048	14.1121	5.172 * 10^(^ − 2)	−4.41176	−4.40116	2.404 * 10^(^ − 3)
5	4	12.4577	12.4714	1.101 * 10^(^ − 1)	−5.88235	−5.88836	1.02 * 10^(^ − 3)
5	5	10.3401	10.2999	3.884 * 10^(^ − 1)	−7.35294	−7.48702	1.823 * 10^(^ − 2)
